# Clothing and Health

**Published:** 1921-03-05

**Authors:** 


### Clothing and Health.

The discussion on the ideal uniform for a nurse, which has been raised by the Nursing Council, is another evidence of the tremendous interest taken by moralists and medical men in the subject of woman's dress. If her clothes are long (as the Paris designers have decided they shall be this season), they sweep up microbe-laden dust, and if they are short the wearer is supposed to die immediately, or later, of pneumonia. We do not think that mortality statistics would show themselves affected much by whatever clothes have been in fashion for women, and in spite of the common notion that women who wear "pneumonia" blouses ought to die of that disease, as a matter of fact in all probability very few of them do so. This is not to justify the wearing of clothes which obviously have the gravest effects on health, but it is useless to ignore other factors in the case. The main fact which is generally ignored is that when women adopt some extreme fashion they soon adopt some antidote to its disadvantages. With the introduction of the low neck comes the fashion for furs. Light clothing is quickly followed by warmer wraps; and the ordinary coats worn by women nowadays would have been regarded as only suitable for a Christmas railway journey some years ago. The long-skirt mode brings in its train the universal fashion of carrying the skirt raised from the ground. Those whose lives are mostly spent in the uniform of formal masculine clothes or of the nurse's usual garb may feel a little contempt for a state of mind which engages so regularly in getting into difficulties for which it has to set about at once finding ways of escape, but it is well that we should realise the facts of the case in the interests of a reasonable attitude towards the question of dress. Because women who wear flimsy clothing do not immediately catch cold and die, no one is entitled to say that flimsy clothing does not deserve the censure passed on it. The truth is that women as a rule escape its dangers by adopting special means, but the fact that the logical effects of careless or foolish dressing are camouflaged in this way ought not to blind us to the real folly of it. Change and variety of dress have their distinct mental value, but there is room for all the variety necessary within the limits of reasonable fashions. It is both foolish and wasteful to wear such thin clothes that extra coal and extra food are necessary to keep up the warmth of the body, just as, on the other hand, it is wasteful to burden the body with a weight of clothes which limit its activity and efficiency. It is reported that certain French Government offices have forbidden the wearing of extravagant clothes to their women officials. Reform does not lie this way. Desire, checked in one direction, will break out more strongly in another. The real progress towards sense and sensibility will come by the spread of true and rational ideas on the subject as a whole.

